# A Multi-Level Geographical Study of Italian Political Elections from Twitter Data

**DOI:** 10.1371/journal.pone.0095809

**Published:** 2014-05-06

**Authors:** Guido Caldarelli, Alessandro Chessa, Fabio Pammolli, Gabriele Pompa, Michelangelo Puliga, Massimo Riccaboni, Gianni Riotta

**Affiliations:** 1 IMT Institute for Advanced Studies, Lucca, Italy; 2 Istituto dei Sistemi Complessi (ISC), Department of Physics, Università “Sapienza”, Roma, Italy; 3 London Institute for Mathematical Sciences, London, United Kingdom; 4 Linkalab, Complex Systems Computational Laboratory, Cagliari, Italy; 5 Center for Polymer Studies and Department of Physics, Boston University, Boston, Massachusetts, United States of America; 6 Department of Managerial Economics, Strategy and Innovation, Katholieke Universiteit Leuven, Leuven, Belgium; 7 Department of French and Italian, Princenton Univerity, Princeton, New Jersey, United States of America; University of Maribor, Slovenia

## Abstract

In this paper we present an analysis of the behavior of Italian Twitter users during national political elections. We monitor the volumes of the tweets related to the leaders of the various political parties and we compare them to the elections results. Furthermore, we study the topics that are associated with the co-occurrence of two politicians in the same tweet. We cannot conclude, from a simple statistical analysis of tweet volume and their time evolution, that it is possible to precisely predict the election outcome (or at least not in our case of study that was characterized by a “too-close-to-call” scenario). On the other hand, we found that the volume of tweets and their change in time provide a very good proxy of the final results. We present this analysis both at a national level and at smaller levels, ranging from the regions composing the country to macro-areas (North, Center, South).

## Introduction

The recent growth of social networks has created various communities of millions of users in constant evolution. It is fair to say that the present widespread access to the Internet is changing the way in which people interact, exchange information and communicate, resulting in a profound transformation of our society and our economy. Differently from other societal changes in the past, we are now able to track down the onset and evolution of this revolution by inspecting the big-data records of social media. Not surprisingly then, many studies from different field perspectives are trying to understand how to deal with this new social phenomenon and how to extract information from the continuously growing repositories [1], [2]. Social science, statistical physics, computer science and network theory methods are being used by scientists to deal with these big data analyses [3]. Irrespective of the technical differences and their usage, the various social networks like Facebook, Twitter and Google+, and search engines like Google and Bing, have relevant information stored in their stream of data [4].

For example, the analysis of query logs from search engines and analysis of Twitter anticipates the spreading of the flu [5]–[8] (simple checks of this phenomenon can be tested with the Google service Flutrends - http://www.google.org/flutrends/ - where data are aggregated on a weekly timescale. Note that the issue of actual prediction has been recently challenged [9]). Similarly query logs anticipate stock-trading volumes [10]. Facebook data reveals the opinions, choices and tastes (and therefore the community structure) of people [11]. Analysis of the growth of Wikipedia quantifies strength of correlation and cultural similarities/differences [12], [13]. In the same spirit, we use data from the microblogging platform Twitter to investigate people's ideas and sentiments [14]. Twitter is a platform where anyone can write posts, with a maximum length of 140 characters, known as “tweets”, which show up in their followers' feeds (a “follow” on Twitter does not equate to a symmetrical relationship). Nowadays, Twitter is used by journalists as the online media of choice to propagate information, and accordingly most of the political debates take place on this social network. The general interest in these new media now makes it possible to accumulate information about social habits, allowing for the unprecedented possibility to analyze social phenomena while keeping perturbation as small as possible. This kind of analysis outperforms traditional polls in three aspects. Firstly, Twitter analysis can essentially be done in real time due to the intrinsic digital form of the data. Secondly, thanks to the success of the service, the numbers of participants are typically orders of magnitude larger than those in traditional polls. Thirdly, (as in the case of this analysis) big data have been proved more reliable than any other way of collecting information. It is conceivable that social media users do not think that their actions will actually be monitored and analyzed. Therefore, it is fair to assume that they are likely to act spontaneously on the web. Similarly to what happens on search engines (where it is pointless to lie), many users on these platforms openly reveal their ideas and interests. Given the above reasons, many research groups have started to study the data stream produced by Twitter. Despite the fact that a fraction of tweets is due to the automatized activity of robots, a significant part of the Twitter content is still produced by genuine users discussing social and general interest issues [15], [16]. It is then possible therefore to detect trends and movements as they are generated in the public sphere of TV shows [17], at the onset of political activity [18]–[23] and in the development of public sentiment in general [24]. Even if some scholars question the validity of this approach [25], [26], it is easy to predict that forecasting studies will become increasingly frequent. Regarding using the analysis of Twitter to forecast the result of political elections, we found a variety of different results.

In one case of study, the ranking of the time series of tweets reproduces the same ranking observed in the elections [27]. Other authors [28] find that both the tweets' time series (volume) and their change in slope may be used as proxies for the elections results (in any case the agreement grows as the election day approaches). In another paper, the authors perform sentiment analysis by means of a semantic scrutiny of the tweets [29]. In this case the forecast regards the 2008 US presidential elections. In the best choice of a 15-day window, the authors find a correlation coefficient between polls and tweets with “Obama” up to r  =  79% (it must also be noted that tweets with the name of the other candidate “McCain” correlate with r = 74% to higher Obama ratings in the polls). Finally, in a study on recent US elections [30], the time series of mentions of various Republican candidates correlates with the difference between the number of votes they received with respect to the Democratic candidates. Overall, a clear indication of correlation (not to mention causality) between quantity and tweets must still be identified. This is possibly due to the intrinsic societal differences in all of the above cited scenarios. Nevertheless, nobody has investigated whether the forecasts (if present) are robust with respect to the change of scale in these investigations. Does the consensus build up above a certain number of users? Is the digital divide large enough amongst both more and less wealthy regions of a country to make social media an unreliable source of information? To answer these questions and understand the passage from the microscopical scale of users to the macroscopic scale of society tendencies, we consider here the various behaviors we observe passing from the national to the regional scale in the aggregation of microscopical data. In this paper we present a multi-scale analysis of the Twitter evolution in the period before the Italian elections of 2013. Data correspond to one month of activity - or 3,378,442 tweets - that we monitored in real time. To our knowledge, this is one of the first in-depth analyses of a political election in Italy to utilize a real-time social media platform like Twitter. All of the collected data were first aggregated into the regions in which Italy is administratively divided. There is not necessarily a one-to-one correspondence between the region declared by the user and the electors in that region. More generally, there is no one-to-one correspondence between Twitter users and electors. Nevertheless, we show in the following section that quantities related to Twitter volume are a good proxy of the electors' behavior. We filtered the tweets by looking at particular terms, namely user identity (terms starting with the symbol “@” called ‘mentions') and hashtags (special keywords in the tweets, indicated by a term after the symbol “#”). In particular, we collected the tweets related to the names of the principal contestants in the elections (considering all the possible aliases we found in a preliminary survey). Indeed, in Italian elections it has become quite commonplace to identify parties with the names of their leaders. This association is so strong in fact that some of the parties involved in the election incorporate the name of the leader into their logo (even if their leader does not run in the election). Among the main results of this investigation, we determined that we can monitor the strong presence of three different political parties (one of them, the “Movimento 5 Stelle”, obtained an unexpectedly good results in the elections) when considering the volume of tweets and their leader's name. Secondly, we obtained an approximate forecast of the relative strength of one party against another by measuring the ratio of their temporal changes cumulated volume in the days immediately before the vote. Such predictive power becomes decreasingly precise when the aggregation scale goes down. By using this method at a national level we can recover the approximate rank of parties in the elections with few exceptions.

## Results

**Figure 1:**
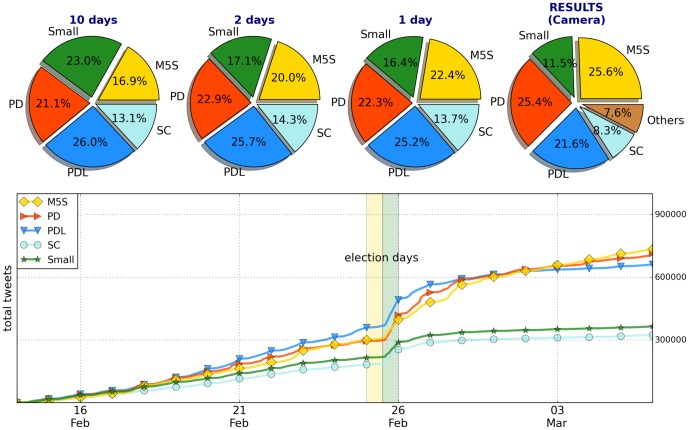
Results of the elections and Twitter volumes. On top: the pie-chart of the results obtained in the election by the parties. Then the forecast from the Twitter time series frozen at three different final times. Below: the cumulative number of tweets for the various parties and their daily evolution. The yellow and green vertical lines represent the election day and the day after respectively (when exit-polls are released).

**Figure 2:**
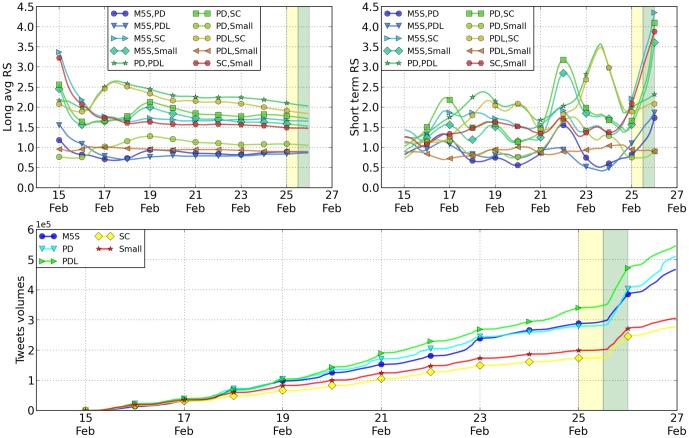
Relative Support from the Twitter volumes, entire country. Top left: the plot of the RS parameter computed on an average of 10 days centered on the specific day on the x-axis; top right: the values of the RS parameter with no averaging day after day. The parties have been ordered according to their final rank at the elections, with the exception of the ratio SC-SMALL. Note how most of the values are above 1, correspondingly SC-SMALL is below one. Below we have a detailed plot of the tweet volume in the final days before the elections, where changes of linear slopes are especially evident during voting days (yellow in all the figures). The yellow and green vertical lines represent the election day and the day after respectively (when exit-polls are released).

We followed the state of the art approach in the analysis of the Twitter data. That is, we considered both the volume of the tweets and the ratio of the time variation of this volume when considering two different parties. The presence of candidates/parties in the press is believed to be a good indicator of the rank they will achieve in the elections. The extension of this feature to Facebook [31] and Twitter has been attempted, but the number of studies on this topic is not large enough to obtain a clear indication. Regarding the time series of tweets, some studies seem to confirm that Twitter [27], [30] can be used as a good proxy for election results. In terms of the ratio of volume variation, we used a newly introduced approach known as the Relative Support (RS) parameter. The RS is an instant indicator of the comparative strength of two political parties - A and B - in Twitter, and it is given by 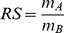
(1)where *m_A_* and *m_B_* are the slopes for the accumulated mentions of the A and B political parties. This formula was claimed to have been particularly useful in predicting the results in Spanish elections [28]. As shown below, which is also true for the study presented here, we find some degree of prediction power, (although it decreases as the scale becomes lower). Figure 1 shows a comparison between the volume of tweets for the various parties and the total number of votes received from electors within in the Italian territory in one of the two branches of the parliament (the “Camera dei Deputati”; for the electoral system at work, see in the Supplementary Information). In our analysis we considered four main parties: (a) the “Partito Democratico” (PD), a center-left party for which we studied the tweets with “Bersani” and “Renzi”; (b) the “Popolo della Libertà” (PDL) a center-right party for which we considered the tweets with “Berlusconi”; (c) the “Scelta Civica” (SC) party for which we followed the tweets with “Monti”; (d) the “Movimento 5 Stelle” (M5S) a political movement for which we followed the tweets with “Grillo”. We also followed several smaller parties that we aggregated below under the name “Small”. They are: the “Sinistra Ecologia and Libertà” (Vendola); the “Lega” (Maroni); the “Unione di Centro” (Casini); “Fare Futuro” (Fini) and the “Fratelli d'Italia” (La Russa, Meloni). Within these elections, which were essentially too close to call, two parties (the M5S and the PD) received more or less the same number of votes, although the M5S received more votes from electors within the Italian territory, while the PD received more when considering Italian citizens living abroad. This scenario was replicated for the other House of Representatives (the “Senato”), which witnessed a draw between the parties. The results regarding the number of votes and tweets are shown in Figure 1 where we present the time evolution of tweet volumes. The yellow and green vertical lines represent the election day and the day after respectively (when exit-polls are released); all the data are summarized in Table 1. As can be seen from the figures, the number of tweets is not a deterministic predictor of election winners; at most, it gives a good indication of the trends present. In these elections we saw three parties (PD, M5S, PDL) separated by just a few percentage points, representing therefore a classic too close to call case. Nevertheless, as the date of the election approaches, the percentage of tweets becomes increasingly accurate with the M5S and PDL more or less at the same level, with a slightly better positioned PDL. A more refined indicator that has been proposed [28] is the previously defined RS parameter, which considers the changes of slopes of the cumulative volumes for specific parties. The resulting quantity is considered to be a forecast of the relative success of one party with respect to another one. Figure 2 shows the behavior of this function at different times; the two vertical stripes indicate the day of and day after the elections, after the results begin to be released. In Table 2 we summarize the values of such ratio. The RS is computed on an ensemble of 192 points starting retrospectively at two days before the election day. Every point corresponds to one hour, so that the whole period analyzed goes from 2 to 10 days before the elections. To reproduce the rank of the ratio of votes, all the values listed in the order above should be larger than one. It can also be observed, in Figure 2, that the fluctuations of the RS parameters are quite large in the period considered. In Figure 2a and 2b we present the daily value obtained with two different averages. Below, in Figure 2c, we include detailed view of the changes in the slope of the tweet volume in the final days. As per the values presented in Table 2, we see that the agreement in the figures is not perfect. Nevertheless, without anticipating the real number of votes, this parameter captures the relative rank of the different parties. To make visual inspection easier, we have computed the RS parameter by ordering the parties with the rank they obtained in the real elections. In such a way, whenever the RS parameters are larger than one, they correctly predict the real outcome. The only exception to that rule is the couple made up by the “SC” party and the parties classified within “OTHERS”, where the former obtained less votes than the latter and the RS in this case is correctly lower than one. Data from Twitter can be tracked down to the level of the city that the user declared during registration. Data from elections is available at the regional (or subdivision) level. We aggregated the data for both levels considering Italy's three main macro-areas (North, Center, South). The areas we considered are based on the definition in the Nomenclature of Territorial Units for Statistics, from Eurostat, that is

**Table 1:** The second column reports the official data from the Italian Interior Minister; these data, taken from http://elezionistorico.interno.it/index.php?tpel=C, have been attached in the Supplementary Information.

Party	Votes	Votes (%)	*T* ^1^ (%)	*T* ^2^ (%)	*T* ^10^ (%)
M5S	8,691,406	25.26	22.4	20.0	16.9
PD	8,646,034	25.43	22.3	22.9	21.1
PDL	7,332,134	21.56	25.2	25.7	26.0
SC	2,823,842	8.30	13.7	14.3	13.1
Small	3,914,229	11.51	16.4	17.1	23.0
Others	2,598,110	7,64	–	–	–

The total number of voters is 35,270,926 from which we have to remove 1,265,171 not “valid votes” (359,279 of which left blank). The percentage are then computed on the total of 34,005,755 “valid” votes. All the above figures do not take into consideration the region “Valle d'Aosta”. As reported above in “Small” we aggregated the votes of the Lega, Futuro e Libertà, Unione Di Centro, Fratelli d'Italia, Sinistra Ecologia e Libertà. For this reason the numbers do not equal the total number of votes, nor do the percentages add up to 100 but rather to 92.34. In other words, other minor parties are missing and have been not monitored on Twitter. From the third column we present the percentage of votes and then the percentage of tweets at 1, 2 and 10 days before elections.

**Table 2:** The error is the statistical error on the various values computed.

Parties A,B	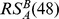	Ratio of Votes
M5S-PD	**1.1±0.3**	1.0052
M5S-PDL	**0.9±0.3**	1.1850
M5S-SC	1.8±0.5	3.0769
M5S,Small	1.6±0.5	2.2208
PD-PDL	**0.8±0.2**	1.1789
PD-SC	1.7±0.1	3.0610
PD-Small	1.5±0.1	2.2093
PDL-SC	2.0±0.2	2.5966
PDL-Small	1.8±0.3	1.8741
SC-Small	**0.9±0.1**	0.7217

A correct prediction means that the ratio of votes and the RS should be either less or more than 1. We note that this parameter recovers all the the ranks with the exception of M5S-PDL, PD-PDL where no prediction is given at any rate because within the error the ratio can be both larger and smaller than one. We indicate this exception with a bold font number.

- **Northern Italy** Piedmont, Lombardy, Liguria, Trentino Alto-Adige/South Tyrol, Veneto, Friuli Venezia Giulia, Emilia Romagna (Vallée d'Aoste/Aosta Valley has been removed from analysis)
- **Central Italy** Tuscany, Marche, Umbria, Lazio.
- **Southern Italy + Islands** Abruzzo, Molise, Campania, Basilicata, Apulia, Calabria, Sicily, Sardinia.

**Figure 3:**
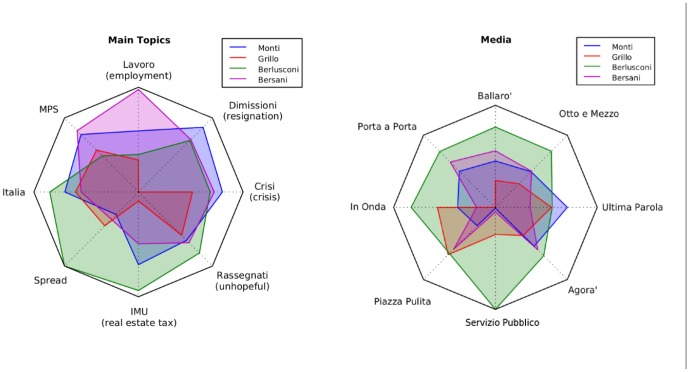
Spider plot of TV shows, and topic relevance for each candidate. In this figure we show two spider plots related to the candidates and two main hashtag categories: TV shows and the main terms used in the political discussion shared by all the candidates. Amongst those we selected the 8 most popular. The number of tweets obtained by each candidate associated with a specific hashtag are reported on a logarithmic scale on the relative axis. Mr. Berlusconi outperforms all the others in the TV show section, while in the other case the concepts and words attached to Mr. Bersani (left-wing party) are not that different from those related to Mr. Monti (moderate party), with the notable exception of the word “lavoro” (employment). In both cases the spider profile of Mr. Grillo is sensibly different from all the others.

**Figure 4:**
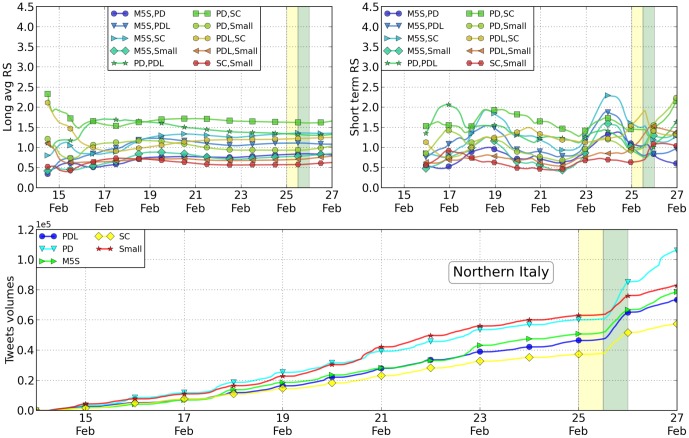
Relative Support from the Twitter volumes, Northern Italy. For the case of the the macro-area Northern Italy, top left: the plot of the RS parameter computed on an average of 10 days centered on the specific day on the x-axis; top right: the values of the RS parameters with no averaging day after day. The parties have been ordered according to their final rank at the elections at the national scale. Since parties ranking differ locally, some of the plots are actually below 1. Below we have a detailed plot of the tweet volume in the final days before the elections, where changes of linear slopes are especially evident during voting days (yellow in all the figures).

**Figure 5:**
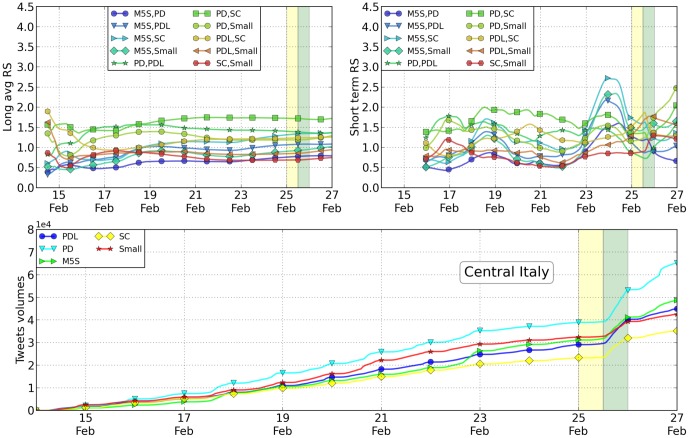
Relative Support from the Twitter volumes, Central Italy. For the case of the the macro-area Central Italy, top left: the plot of the RS parameter computed on an average of 10 days centered on the specific day on the x-axis; top right: the values of the RS parameters with no averaging day after day. The parties have been ordered according to their final rank at the elections at the national scale. Since parties' ranking differ locally, some of the plots are actually below 1. Below we have a detailed plot of the tweet volume in the final days before the elections, where changes of linear slopes are especially evident during voting days (yellow in all the figures).

**Figure 6:**
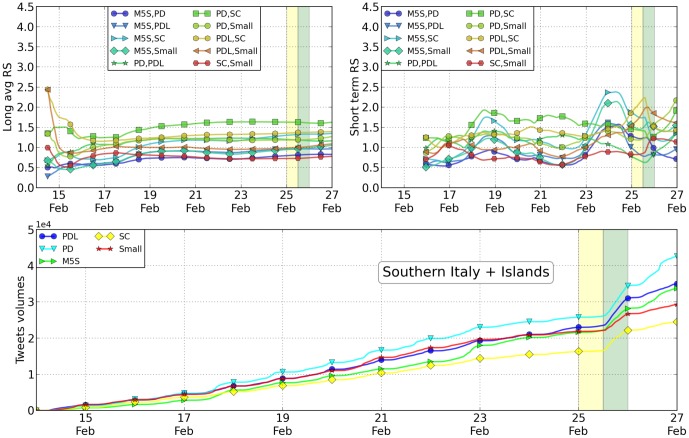
Relative Support from the Twitter volumes, Southern Italy and Islands. For the case of the the macro-area Southern Italy and Islands, top left: the plot of the RS parameter computed on an average of 10 days centered on the specific day on the x-axis; top right: the values of the relative supports with no averaging day after day. The parties have been ordered according to their final rank at the elections at the national scale. Since parties' ranking differ locally, some of the plots are actually below 1. Below we have a detailed plot of the tweet volume in the final days before the elections, where changes of linear slopes are especially evident during voting days (yellow in all the figures).

In Tables 3 and 4 and Figures 3, 4 and 5, we reported the analysis of tweets versus votes for these three areas. Except for only a few cases, the results are as accurate as those at the national level. As shown in the Supplementary information such accuracy decreases by reducing even more the scale of analysis. As previously mentioned, the RS values have been computed daily by averaging the 24 values measured hourly within the same day (short average) or by averaging the 48 values measured hourly the same day and the day before in a one month period. The error reported in Table 2 is the standard deviation of the data. Despite the fact that the geographical distribution of the votes is different in the Italian territory, these results seem to be rather robust. Traditionally, as is the case withthese elections as well, the Northern part of Italy leans towards the Lega (a political party classified as “Small” in our analysis), while the Central part is left-wing and the Southern part is more conservative. Such behavior is reflected in the Twitter volumes when they are disaggregated on a geographical basis. In particular, in these three macro-areas the winning party is different and the Twitter analysis in fact correctly reproduces the results. At the smaller regional scale, the statistical error starts to become so large that most of the measurements become inconclusive. The precise figures and analysis are available within the Supplementary Information section attached to this paper. Finally, we tried to characterize the overlap in the messages where the various candidates were present. In this light, we first listed the most popular hashtags associated with the name of the political leaders. Then, we restricted our analysis to the hashtags in this list that have been associated with every candidate at least once. Amongst these terms, we selected the 8 most popular ones. From a sociological point of view, it is probably interesting to mention that those correspond to TV shows where the candidates were present or cited. We then construct a spider plot for every candidate by indicating these hashtags with axes and by measuring on them the number of tweets measured. Such number of tweets obtained by each candidate associated with a specific hashtag is reported on a logarithmic scale on the relative axis as shown in Figure 6. The more two candidates overlap, the more their public image on these shows is similar. Far below in popularity, candidates are also associated to political ideas or concepts. We repeat the same analysis for the 8 most popular concepts. The visual results we get from this analysis may seem familiar to experts of Italian politics. Namely, Mr. Berlusconi has the strongest correlation with tweets on all 8 of the TV shows considered. The concepts and words attached to Mr. Bersani (left-wing party) are not that different from those associated with Mr. Monti (moderate party), with the notable exception of the word “lavoro” (employment). In both cases the spider profile of Mr. Grillo is sensibly different from all the others. A more quantitative measurement of the overlap between candidates is shown in Table 5.

**Table 3:** The second column reports the official data from Italian Interior Minister.

Party	Votes	Votes (%)	T 1d (%)	T 2d (%)	T 10d (%)
**Northern Italy**					
M5S	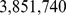	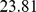	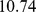	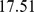	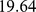
PD	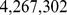	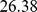	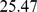	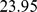	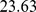
PDL	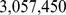	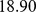	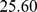	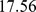	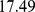
SC	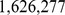	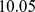	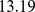	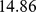	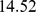
Small	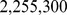	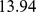	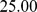	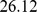	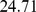
**Central Italy**					
M5S	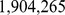	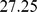	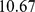	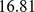	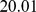
PD	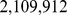	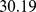	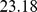	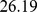	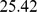
PDL	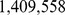	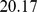	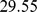	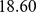	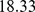
SC	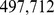	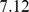	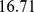	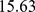	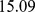
Small	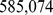	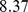	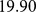	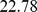	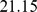
**Southern Italy + Islands**					
M5S	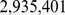	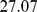	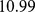	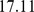	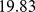
PD	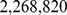	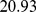	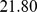	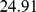	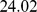
PDL	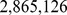	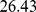	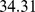	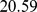	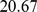
SC	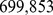	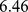	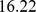	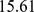	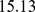
Small	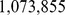	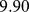	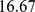	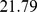	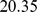

The total number of voters is 16,700,117 (16,174,594 valid) for Northern Italy, 7,218,645 (6,989,394 valid) for Central Italy, 11,352,164 (10,841,767 valid) for Southern Italy and Islands. Twitter values are reported from the third column; in particular the percentage of tweets at 1, 2 and 10 days before elections.

**Table 4:** The RS is computed on an ensemble of 192 points starting retrospectively at two days before the election day.

	Northern Italy		Central Italy		Southern Italy + Islands	
Parties A,B	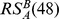	Ratio	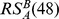	Ratio	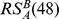	Ratio
M5S,PD	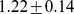	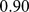	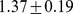	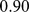	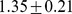	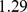
M5S,PDL	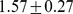	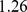	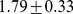	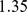	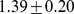	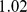
M5S,SC	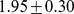	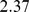	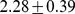	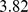	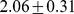	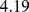
M5S,Small	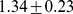	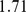	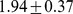	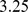	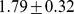	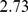
PD,PDL	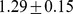	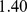	1.31±0.20	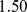	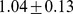	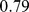
PD,SC	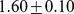	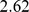	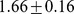	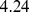	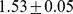	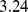
PD,Small	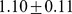	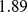	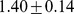	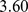	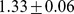	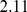
PDL,SC	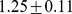	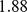	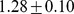	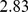	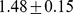	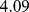
PDL,Small	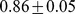	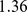	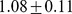	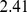	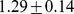	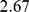
SC,Small	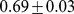	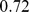	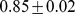	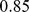	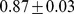	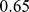

Every point corresponds to one hour, so that the whole period analyzed goes from 10 days before the elections to 2 days before the election. We keep the list of ratios the same as in the main paper, reflecting the strength of parties at a national level, despite the fact that some of these ratios are now different. “Ratio” represents the ratio of votes obtained by the two parties in the first column, i.e. votes(A)/votes(B). Even the numerical agreement with the ratio in terms of votes seems rather good. Only the cells whose font is bond present deviations from expected.

**Table 5:** In this table we present the cosine similarity between all the possible couples of politicians present in the spider plots.

Politicians A,B	Main Topics	Media
Berlusconi-Grillo	0.89	0.86
Berlusconi-Monti	0.91	0.71
Berlusconi-Bersani	0.65	0.67
Grillo-Monti	0.96	0.82
Grillo-Bersani	0.80	0.84
Monti-Bersani	0.88	0.96

The two categories used to compare their behavior are the Main Topics discussed during the election period and the mentions of the most important TV shows related to politics.

## Discussion

Despite the importance of the topic, the state of the art of Twitter analysis of electors' intentions it is still in its infancy. Notwithstanding the clear bias found through the representation of Twitter user statistics with respect to the general population of eligible voters, the information regarding opinion orientation that can be extracted from the platform is still valuable [32]. Even if there is a clear bias in the Twitter use with respect to the general voter population, the information that is possible to get regarding the opinion orientation is still valuable. At the time this article was written, most of the studies published report a significant correlation between Twitter activity and election results [18]–[20], [28]. Others display little or no correlation [25], [26], [29]. Unfortunately, the case-studies reported in the literature are discordant, making a direct comparison simply not possible. For example, in the present study, three different parties got a similar number of votes, and therefore few polls were able to predict the final outcome. Nevertheless, in the analysis we present here, we are able to detect a strong presence of the M5S party in the Twitter space and the relative weakness of the SC. Before knowing election results the two results were largely unexpected.

More in detail, we recovered most of the correct relative strengths of one party against the others by considering the ratio of the slope changes in the cumulate distributions of tweets. This parameter, called the RS parameter, seems to be a fair predictor of the relative strengths of political parties. Two notable exceptions are present in both of the previous analyses. They refer to the PDL and the SC, which both received a larger than expected volume of tweets when considering their electoral result. One possible ‘ex post' explanation may be that the tweets for these two parties were related to “Berlusconi” and “Monti”, who were, respectively, the former and current prime ministers at the time of the elections. Moreover, a noticeable number of the tweets recorded may refer to their public activity rather than to their political opinions. Finally, in the same period Mr. Berlusconi was involved in a series of trials that were extensively covered by the media, which could have been the driving force behind some of the tweets in which he was mentioned. He also made a provocative move regarding the abolition of the IMU (a sort of Council Tax) that was widely discussed throughout the internet and beyond, which probably resulted in a substantial peak in tweet volume. Remarkably, the indications we can obtain from the tweet analysis remain valid even after changing the scale at which the comparison is done. Using personal user profile information, we can link tweets to a specific location to comparethem against the votes in the same area. For the purposes of this analysis we break Italy down into three macro-areas (North, Center and South); the size of these geographic divisions ensures a good statistical outcome. It is worth noting that the ranking of the main parties differs in these three macro-areas with respect to the national one. Nevertheless, the vote-tweet correlation still holds at the macro-area level. The notably good results of the Lega (Small) in the North, and the PD in the Center are confirmed by tweet volume. This paper confirms what has been already found in a series of similar analyses. Twitter data can be an effective way to get indications on the election outcomes. The degree of confidence is bounded by the statistical errors in the data. To limit this problem, for our analysis we collected an unprecedented database of more than 12 million tweets, 3 million of which dating to the period immediately preceding the political elections of February 24*^th^*–25*^th^* 2013. Such a database represents, to our knowledge, one of the largest playgrounds currently available for the analysis of the evolution of elector opinions. It is fair to stress the intrinsic limitations in the search results we had to face when collecting this data once again. We are nevertheless confident from the results that no spurious correlations have been introduced in the sampling procedure. To extend this analysis, we are presently envisaging a more careful description of the bipartite network between hashtags and politicians in order to obtain a better profiling of the political candidates.

## Materials and Methods

The present dataset was collected through the Twitter API which is made available to search its database. We used the Python version called “Twython” to collect more than 12 million tweets from November 22nd to March 15th 2013. In this paper we present the analysis on 3,378,442 tweets from before and during the election days (February 24*^th^*–25*^th^* 2013). A reduced and privacy compliant version of the data is available at the link http://www.linkalab.it/data. Given the fact that Twitter puts a limit on the volume of the search results one can get through the API, this dataset corresponds to a fraction of the real activity on the social platform. This introduces a bias in the sense that queries with a potentially large number of results are underrepresented with respect to queries whose results are lower in volume. The tweets selected were chosen by considering candidate and leader mentions (including all selected aliases) of the various parties (see the Supplementary Information for a detailed list). In particular we focused on the 4 largest (expected) parties: the Movimento 5 Stelle (M5S), the Partito Democratico (PD), the Popolo della Libertà (PDL), and the Scelta Civica (SC). In terms of people:

- “Grillo” for the M5S party
- “Bersani” and “Renzi” for the PD
- “Berlusconi” for the PDL
- “Monti” for the SC
- for Small, the names “Vendola” (Sinistra Ecologia e Libertà), “Maroni” (Lega), “Meloni” (Fratelli d'Italia), “Fini” (Futuro e Libertà), “Casini” (Unione di Centro).

These associations are justified by the fact that the PDL and SC put the name of the candidate on the party's logo, creating a sort of visual representation of the strong connection between the candidate and the party. This sort of personification of the political debate also applies to the other parties. The database has been aggregated both at the hourly level, for the computation of the RS parameter, and at the daily level to measure tweet volume. The information retained for the purpose of this study includes: i) the text of the tweet; ii) the hashtag list; iii) the time stamp; iv) user information like the geographical location (when available), the number followers and those followed, tweet counts; v) type of tweet (i.e. new post, reply, retweet). Data were properly anonymised by masking the user-id with a numerical code. By location we mean the geographical place declared in the profile and not the GPS coordinates linked to the location from where the user sent the tweet. This is more appropriate for two reasons. Firstly, the location in the profile is more likely to be the same as where the elector votes, and secondly very few users let Twitter follow their movements through the GPS tracking system. With respect to the state of the art in the Twitter analysis, we restricted our analysis to three main points. Firstly, we studied the evolution of the number of tweets over time. Secondly, we analyzed the derivative of the number of tweets over time, a quantity defined below as the Relative Support parameter. Finally, we used the tweets to measure the distance of two candidates by means of a semantic analysis of the tweets in which they are cited.

### Time Series

Regarding the time series of activity, i.e. the number of tweets related to the election, we started our analysis one month before the elections. We then considered three different ending points for the series:

- The tweets in the period ending 10 days before elections
- The tweets in the period ending 2 days before elections
- The tweets in the period ending the day of elections

The idea is to determine if the Twitter volume is a good proxy of the final vote, and if so, when this information could be available. A particular activity on the election day - a time series discretized at the hour level showing a linear behavior with a series of changes of slope - has also been observed [28]. Exactly These slopes [28] are at the basis of the RS as described below.

### Relative Support Parameter

The RS parameter is defined for a couple of parties, A and B, as 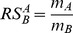
(2)where *m_A_* is the slope of the cumulate number of tweets for party A, and similarly *m_B_* is the slope of the cumulate number of tweets for party B. Note that this slope is the discrete version of the derivatives, i.e. it is a finite difference of volumes of the kind 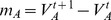
(3)and therefore it does not depend upon the starting time of observation. For such a quantity we discretized the tweets at hourly intervals and we computed its average value along the 24 hours of the day before the election (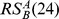) and the day before that (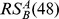).

### Distance of candidates

Another field that can be investigated by means of Twitter analysis is candidate profiling. By considering the keyword with which they are associated in the tweets we can measure their positioning with respect to the political topics, and thereby also measuring their reciprocal distance. More specifically, we rank the keywords associated with the various politicians. As multiple politicians can often be associated with similar keywords, it may be possible to define a subset of shared keywords. At this point every candidate can be represented by a curve on a spider plot where the various axes correspond to one of these keywords. Another approach for measuring the distance between different candidates is to consider how many times they are co-cited in a tweet. The set of all the co-presences forms a complete graph, although the skeleton of the largest correlation can be represented by a minimal spanning tree.

## Supporting Information

Table S1 **Data extracted and aggregated from the following official files from Ministero degli Interni.** The table shows the results of the Italian Elections with the aggregations used in the paper.
(XLS)

Table S2 **Original data file from Ministero degli Interni showing the aggregated results for Camera dei Deputati for the entire country with details of parties and coalitions.**
(CSV)

Table S3 **Original data file from Ministero degli Interni showing the non aggregated, regional level, results for Camera dei Deputati for the entire country with details of parties and coalitions.**
(CSV)

Table S4 **Original data file from Ministero degli Interni showing the non aggregated results for Camera dei Deputati for the entire country without details of parties and coalitions.**
(CSV)

Table S5 **Original data file from Ministero degli Interni showing the aggregated results for Camera dei Deputati for the regions with details of parties and coalitions and without macro-area aggregations (southern/northern/central Italy sums).**
(CSV)

Table S6 **Original data file from Ministero degli Interni showing the aggregated results for Camera dei Deputati for the regions without details of parties and coalitions but with macro-area aggregations (southern/northern/central Italy sums).**
(CSV)
